# The Prevalence and Risk Factors of COVID-Stress Syndrome

**DOI:** 10.7759/cureus.39388

**Published:** 2023-05-23

**Authors:** Ahmad Al Houri, Abdullah Alhouri, Hanaa Zahrawi, Hasan Al Houri, Bilal Abu-Hussein, Douaa Mohammad Nazir Arrouk, Yazan Jarrar, Ahmad Al-Thunaibat, Obada Latifeh, Aiman Al Sharei, Youssef Latifeh

**Affiliations:** 1 Department of Medicine, Syrian Private University, Damascus, SYR; 2 Internal Medicine, Royal Berkshire Hospital, Reading, GBR; 3 Department of Internal Medicine, Damascus University, Damascus, SYR; 4 Department of Internal Medicine, Syrian Private University, Damascus, SYR; 5 Department of Medicine, The University of Jordan, Amman, JOR; 6 Applied Statistics - Quantitative Methods Department, Damascus University, Damascus, SYR; 7 Department of Medicine, King Hussein Cancer Center, Amman, JOR; 8 Faculty of Medicine, Damascus University, Damascus, SYR; 9 Faculty of Medicine, The Hashemite University, Amman, JOR; 10 Department of Psychiatry, Damascus University, Damascus, SYR; 11 Department of Psychiatry, Al Sham Private University, Damascus, SYR

**Keywords:** depression., students, jordanian, syrian, covid-19, css

## Abstract

Background

COVID-19 is a serious disease causing negative psychological effects such as nervousness, isolation, depression, and suicide ideation. The COVID Stress Scale was developed to better understand and assess COVID-19-related distress. University students are predicted to be negatively impacted by the COVID-19 outbreak due to their lack of psychological skills and high levels of academic stress. This study compares the prevalence of COVID stress syndrome (CSS) among university students in Syria and Jordan during the outbreak. The questionnaire used in the study covers multiple aspects and can be applied to future pandemics or infectious diseases.

Methodology

This is a cross-sectional study conducted in Syria and Jordan between September 1 and December 1, 2021, to evaluate CSS among university students. Data were collected from a convenience sample of 2525 students using a structured, validated, and published questionnaire. Ethical considerations were taken, and informed consent was obtained from participants. The questionnaire had two parts: participant characteristics and CSS. The data were analyzed using the Statistical Package for the Social Sciences (SPSS, IBM Corp., Armonk, NY), and the chi-square test was used to compare the CSS scale between the two countries.

Result

The study involved 2525 university students, mostly Syrian (63.6%) and Jordanian (36.4%), aged 18-24 (89.5%), and mostly single (95.6%). Over 50% of students lived in homes with three or more people. More than half reported good to excellent economic status; non-smokers accounted for over 50%. Regarding CSS, 39.8% had a high score, 28% average, 20% severe, and 12.2% low to mild. Jordanian male students and single Syrian students showed a higher probability of experiencing CSS symptoms. The number of people in the household, financial status, and field of study also played a significant role.

Conclusion

The COVID-19 pandemic has negative impacts beyond physical health, including the economy, education, and mental health. A stress scale has been developed to measure COVID-19 stress syndrome, which includes Danger and Contamination Fears (DAN), Socioeconomic Consequences Fears (SEC), Xenophobic Fears (XEN), Traumatic Stress Symptoms (TSS), and Compulsive Checking and Reassurance Seeking (CHE). Non-medical faculty students are more likely to acquire CSS symptoms than medical faculty students.

## Introduction

COVID-19 is a serious disease caused by the severe acute respiratory syndrome coronavirus-2 (SARS-CoV-2), which has been assumed to be a pandemic leading to the deaths of over 2 million people worldwide [1]. This pandemic is considered the most serious international health crisis in our modern era [2]. Nervousness, isolation, stigma, depression, and suicide ideation were among the negative psychological effects of the COVID-19 pandemic [3-5]. According to studies, 25% of the Chinese population suffers from various stages of stress and anxiety, with COVID-19 being a significant contributor [6,7]. For that reason, public health strategies dealing with rapidly growing disease outbreaks such as COVID-19 require a delicate balance between maintaining the public's health and promoting precautionary measures, which, in turn, can lead to emotional and behavioral problems [8]. The concept of COVID-19 in psychiatry has quickly grown. At first, it was considered a form of phobia ("corona phobia") or a vaguely described anxiety-related syndrome [9]. However, observations from previous pandemics suggest that the scope of distress-related symptoms is much broader [8]. Therefore, the COVID Stress Scales were developed to better understand and assess COVID-19-related distress [8]. The CSS has five dimensions: Danger and Contamination Fears (DAN), Socioeconomic Consequences Fears (SEC), Xenophobic Fears (XEN), Traumatic Stress Symptoms (TSS), and Compulsive Checking and Reassurance Seeking (CHE) [10]. Additionally, the five dimensions have demonstrated good to outstanding reliability and validity, with internal consistency (Cronbach alpha) coefficients for each scale being >0.80 and intercorrelated amongst the scales [11]. Students in higher education have significant levels of academic stress and are sensitive to psychosocial issues, yet they lack the psychological abilities to handle their psychological and academic obligations [12,13]. Thus, the COVID-19 outbreak is predicted to have a negative impact on university students' physical and mental health. According to a recent report, university students experienced posttraumatic stress disorder (PTSD), fear, sadness, nervousness, and emotional disorders during the COVID-19 epidemic [6,14].

This study aims to assess and compare the prevalence of COVID stress syndrome (CSS) among university students in Syria and Jordan during the COVID-19 outbreak.

## Materials and methods

Study design

This is a cross-sectional study that was conducted in Syria and Jordan to evaluate CSS among university students. Data were collected between September 1 and December 1, 2021. Undergraduate university students aged 18 years and older who signed informed consent were eligible to be included.

Ethical considerations

This study was approved by the institutional review board (IRB) of Damascus University, Syrian Private University (SPU), and Hashemite University (No. 9/14/2020/2021). The questionnaire included comprehensive information about the study's goals. Informed consent was obtained from participants who volunteered to complete the questionnaire. The data's anonymity and confidentiality were secured by providing each participant with an identification number visible to the research team.

Data collection and measures

Data were collected from a convenience sample of 2525 students using a structured, validated, and published questionnaire [11]. University students at participating universities were given a detailed explanation of the study. The researchers distributed the questionnaire directly to the students. Each questionnaire came with a cover letter that explained the study, its goals, and how to fill it out and return it. The G Power 3.1 software (Düsseldorf University, Westphalia, Germany) was used for prior statistical power analysis for sample size estimation, with a two-tailed alpha of 0.05 and a power level of 0.5 [15]. Using Cohen’s criteria [16], the analysis revealed that a sample size of approximately 768 was needed. Thus, the sample size of 2525 participants included in this study was more than adequate.

The questionnaire included two main parts: the first part consisted of questions regarding participant characteristics, including age in years, gender, marital status, country of study, living condition, number of people living with the respondent, having a job or not, financial status, educational level, do the parents work in the medical field, studying field, smoking, and has the participant been infected by the virus before? The second part of the questionnaire consisted of CSS. During pandemics, the CSS was created to evaluate and assess COVID-19-related distress and health-related anxiety [14]. The scale includes 36 items, which are categorized into five domains: (1) DAN and CON (12 items), (2) SEC (6 items), (3) XEN (6 items), (4) TSS (6 items), and (5) CHE (6 items). Items in the DAN, CON, SEC, and XEN domains are rated on a 5-point scale ranging from 0 (not at all) to 4 (extremely). Similarly, the TSS and CHE domains are scored from 0 (never) to 4 (almost always). The sum of ratings for each item in each domain is used to determine the scores for each of the five domains. To interpret the results obtained, higher scores reflect more intense or frequent perceptions experienced within the last seven days. The total scores were categorized as follows: low distress (<5), mild distress (5-16), average distress (17-36), high distress (37-71), or severe distress (>72).

Statistical analysis

The data were analyzed using the Statistical Package for the Social Sciences (SPSS) software, version 25.0 (IBM Corp., Armonk, NY). Categorical variables were presented as numbers and percentages. The chi-square test was used to compare the CSS scale between the two countries. The statistical significance level was set at 0.05.

## Results

A total of 2525 university students were recruited to participate in the study, with Syrian students accounting for 63.6% (1606) and Jordanian students accounting for the remaining 36.4% (919). The majority of participants (89.5%) were between the ages of 18 and 24. The Jordanian and Syrian samples showed similar percentages of participants between the ages of 18 and 24, with 89.8% and 89.3%, respectively. As regards gender, females were represented (51.7%), while males accounted for 48.3% of the total participating students. Jordanian participants were primarily male (53.1%), whereas Syrian participants were mostly female (54.5%). Regarding their marital status, Jordanian and Syrian students had comparable percentages of unmarried students, who comprise the vast majority of the data, with unmarried Syrian students representing 96.3% and unmarried Jordanian students representing 94.3%. The overall percentage of unmarried students in both nations was 95.6% (Table 1).

**Table 1:** Sociodemographic characteristics.

Variable (N=2525)		N (%)
Age	18–24	2259 (89.5%)
	25–30	218 (8.6%)
	>30	48 (1.9%)
Gender	Male	1219 (48.3%)
	Female	1306 (51.7%)
Marital status	Unmarried	2413 (95.6%)
	Married	112 (4.4%)
Country of studying	Syria	1606 (63.3%)
	Jordan	919 (36.4%)
living condition	Owned house	1749 (69.3%)
	Rented house	638 (25.3%)
	University campus	138 (5.5%)
How many people live in your household?	Alone	123 (4.9 %)
	1, 2	373 (14.8%)
	3, 4	954 (37.8%)
	>5	1075 (42.6%)
The job	I don’t work	2093 (82.9%)
	I have a job	432 (17.1%)
Financial status	Low	116 (4.6%)
	Medium	712 (28.2%)
	Good	1404 (55.6%)
	Excellent	293 (11.6%)
Educational level	Institute degree	30 (1.2%)
	University degree	2424 (96%)
	Master degree	60 (2.4%)
	Doctorate	11 (0.4%)
Parents working in a medical field	No	2074 (82.1%)
	Yes, one of them	352 (13.9 %)
	Yes, both of them	99 (3.9 %)
Studying field	Medical colleges	1193 (47.2%)
	Non-medical colleges	1332 (52.8%)
Smoking	Smoker	1041 (41.2%)
	Non-smoker	1484 (58.8%)

More than half of the students lived in privately owned houses in Syria (71.7%) and Jordan (65%). On the other hand, the Jordanian and Syrian students living on campus had the lowest percentage, with 5.8% and 4.9%, respectively. Both Jordanians and Syrians lived with three or more people in the household, with around 80% of each country's sample. Concerning the economic status, 82.9% of those surveyed were unemployed, of which unemployed Jordanian students made up 79.8% and unemployed Syrian students accounted for 84.7% of the total sample in each country. Good to excellent economic status was reported by more than half of the students in both countries. The medical background was also examined, and the current study revealed that 82% of participants had no parents working in any medical field, a percentage that was divided between both countries by 75.5% for Jordanian students and 85.9% for Syrian students. In contrast, 47.2% of students were studying in medical schools and medical faculties, representing the highest percentage of surveyed students, with around 40% for both countries. Regarding smoking, more than half of the Jordanians and Syrians were non-smokers, with a percentage of 51.9% and 62.7%, respectively (Table 1).

As shown in Table 2, the majority of students in both nations were infected with COVID-19. It is worth mentioning that 52.1% of Syrian respondents had previously been infected with the virus, although it was not confirmed by polymerase chain reaction (PCR).

**Table 2:** Students' response to whether they had previously been infected with the virus.

Categories (N=2525)		Syria	Jordan	Total
Have you been infected with the virus before?	No, I have not	695 (43.3%)	424 (46.1%)	1119 (44.3%)
	Yes, I have had the symptoms, but I have not confirmed it with the PCR	836 (52.1%)	134 (14.6%)	970 (38.4%)
	Yes, I have had the symptoms and I have confirmed it with the PCR	67 (4.2%)	329 (35.8%)	396 (15.7%)
	Yes, I have confirmed it with the PCR, but I have been asymptomatic	8 (0.5%)	32 (3.5 %)	40 (1.6%)

The second part of the questionnaire assessed CSS symptoms and revealed the following CSS scores: 39.8% (1004) had a high score, 28% (708) had an average score, 20% (505) had a severe score, and 12.2% (308) had a low to mild score. The CSS scores are presented in Table 3.

**Table 3:** The total scores of COVID-19 stress syndrome.

CSS scores	N (%)
Low	75 (3%)
Mild	233 (9.2%)
Average	708 (28%)
High	1004 (39.8%)
Severe	505 (20%)
Total	2525 (100%)

Various significant correlations were found between sociodemographic characteristics and CSS scores among Jordanian and Syrian students (Table 4). For instance, there is a significant correlation between age and CSS among Jordanian students, with students between the ages of 18 and 25 having the highest risk of developing CSS symptoms (89.8%), compared to students between the ages of 25 and 30 (7.9%) or those older than 30 (2.3%). Among Syrian students, however, there is no significant association between age and CSS.

**Table 4:** Sociodemographic characteristics.

Variable (N=2525)		CSS	Syria N (%)	P-value	Jordan N (%)	P-value
Age	18–24	Low	45 (97.8%)	0.415	27 (93.1%)	0.22
		Mild	134 (87.6%)		68 (85%)	
		Average	464 (89.9%)		169 (88%)	
		High	627 (89.1%)		262 (87.3%)	
		Severe	164 (87.7%)		299 (94%)	
	25–30	Low	1 (2.2%)		0 (0%)	
		Mild	14 (9.2%)		11 (13.8%)	
		Average	46 (8.9%)		17 (8.9%)	
		High	66 (9.4%)		30 (10%)	
		Severe	18 (9.6%)		15 (4.7%)	
	>30	Low	0 (0%)		2 (6.9%)	
		Mild	5 (3.3%)		1 (1.3%)	
		Average	6 (1.2%)		6 (3.1%)	
		High	11 (1.6%)		8 (2.7%)	
		Severe	5 (2.7%)		4 (1.3%)	
Gender	Male	Low	25 (54.3%)	0.000	15 (51.7%)	0.007
		Mild	88 (57.5%)		49 (61.3%)	
		Average	257 (49.8%)		103 (53.6%)	
		High	296 (42.0%)		177 (59%)	
		Severe	65 (34.8%)		144 (45.3%)	
	Female	Low	21 (45.7%)		14 (48.3%)	
		Mild	65 (42.5%)		31 (38.8%)	
		Average	259 (50.2%)		89 (46.4%)	
		High	408 (58%)		123 (41%)	
		Severe	122 (65.2%)		174 (54.7%)	
Marital status	Unmarried	Low	45 (97.8%)	0.071	26 (89.7%)	0.489
		Mild	149 (97.4%)		76 (95%)	
		Average	503 (97.5%)		180 (93.8%)	
		High	675 (95.9%)		280 (93.3%)	
		Severe	174 (93%)		305 (95.9%)	
	Married	Low	1 (2.2%)		3 (10.3%)	
		Mild	4 (2.6%)		4 (5%)	
		Average	13 (2.5%)		12 (6.3%)	
		High	29 (4.1%)		20 (6.7%)	
		Severe	13 (7%)		13 (4.1%)	
Living condition	Owned house	Low	38 (3.3%)	0.139	18 (62.1%)	0.917
		Mild	106 (9.2%)		57 (71.3%)	
		Average	368 (31.9%)		126 (65.6%)	
		High	497 (43.1%)		190 (63.3%)	
		Severe	143 (12.4%)		206 (64.8%)	
	Rented house	Low	8 (17.4%)		10 (34.5%)	
		Mild	42 (27.5%)		19 (23.8%)	
		Average	117 (22.7%)		55 (28.6%)	
		High	157 (22.3%)		49 (31.3%)	
		Severe	37 (19.8%)		99 (31.1%)	
	University campus	Low	0 (0%)		1 (3.4%)	
		Mild	5 (3.3%)		4 (5%)	
		Average	31 (6%)		11 (5.7%)	
		High	50 (7.1%)		16 (5.3%)	
		Severe	7 (3.7%)		13 (4.1%)	
How many people live in your household?	Alone	Low	6 (13%)	0.243	3 (10.3%)	0.000
		Mild	8 (5.2%)		3 (3.8%)	
		Average	25 (4.8%)		17 (8.9%)	
		High	30 (4.3%)		13 (4.3%)	
		Severe	11 (5.9%)		7 (2.2%)	
	1, 2	Low	7 (15.2%)		3 (10.3%)	
		Mild	28 (18.3%)		8 (10%)	
		Average	95 (18.4%)		28 (14.6%)	
		High	120 (17%)		35 (11.7%)	
		Severe	24 (12.8%)		25 (7.9%)	
	3, 4	Low	13 (28.3%)		7 (24.1%)	
		Mild	55 (35.9%)		24 (30%)	
		Average	207 (40.1%)		79 (41.1%)	
		High	283 (40.2%)		102 (34%)	
		Severe	86 (46%)		98 (30.8%)	
	>5	Low	20 (43.5%)		16 (55.2%)	
		Mild	62 (40.5%)		45 (56.3%)	
		Average	189 (36.6%)		68 (35.4%)	
		High	271 (38.5%)		150 (50%)	
		Severe	66 (35.3%)		188 (59.1%)	
The Job	I don’t work	Low	41 (89.1%)	0.883	19 (65.5%)	0.270
		Mild	128 (83.7%)		62 (77.5%)	
		Average	440 (85.3%)		156 (81.3%)	
		High	592 (84.1%)		236 (78.7%)	
		Severe	159 (85%)		260 (81.8%)	
	I have a job	Low	5 (10.9%)		10 (34.5%)	
		Mild	25 (16.3%)		18 (22.5%)	
		Average	76 (14.7%)		36 (18.8%)	
		High	112 (15.9%)		64 (21.3%)	
		Severe	28 (15%)		58 (18.2%)	
Financial status	Low	Low	1 (2.2%)	0.157	3 (10.3%)	0.000
		Mild	8 (5.2%)		2 (2.5%)	
		Average	20 (3.9%)		3 (1.6%)	
		High	43 (6.1%)		9 (3%)	
		Severe	15 (8%)		12 (3.8%)	
	Medium	Low	9 (19.6%)		12 (41.4%)	
		Mild	43 (28.1%)		27 (33.8%)	
		Average	164 (31.8%)		45 (23.4%)	
		High	229 (32.5%)		82 (27.3%)	
		Severe	69 (36.9%)		32 (10.1%)	
	Good	Low	29 (63%)		9 (31%)	
		Mild	89 (58.2%)		37 (46.3%)	
		Average	278 (53.9%)		104 (54.2%)	
		High	371 (52.7%)		180 (60%)	
		Severe	86 (46%)		221 (69.5%)	
	Excellent	Low	7 (15.2%)		5 (17.2%)	
		Mild	13 (8.5%)		14 (17.5%)	
		Average	54 (10.5%)		40 (20.8%)	
		High	61 (8.7%)		29 (9.7%)	
		Severe	17 (9.1%)		53 (16.7%)	
Parents working in a medical field	No	Low	38 (82.6%)	0.438	23 (79.3%)	0.560
		Mild	129 (84.3%)		64 (80%)	
		Average	442 (85.7%)		148 (77.1%)	
		High	606 (86.1%)		225 (75%)	
		Severe	165 (88.2%)		234 (73.6%)	
	Yes, one of them	Low	8 (17.4%)		4 (13.8%)	
		Mild	19 (12.4%)		11 (13.8%)	
		Average	53 (10.3%)		36 (18.8%)	
		High	75 (10.7%)		64 (21.3%)	
		Severe	20 (10.7%)		62 (19.5%)	
	Yes, both of them	Low	0 (0%)		2 (6.9%)	
		Mild	5 (3.3%)		5 (6.3%)	
		Average	21 (4.1%)		6 (4.2%)	
		High	23 (3.3%)		11 (3.7%)	
		Severe	2 (1.1%)		22 (6.9%)	
Studying field	Medical colleges	Low	17 (37%)	0.000	13 (44.8%)	0.000
		Mild	81 (52.9%)		42 (52.5%)	
		Average	285 (55.2%)		123 (64.1%)	
		High	341 (48.4%)		116 (38.7%)	
		Severe	66 (35.3%)		109 (34.3%)	
	Non-medical colleges	Low	29 (63%)		16 (55.2%)	
		Mild	72 (47.1%)		38 (47.5%)	
		Average	231 (44.8%)		69 (35.9%)	
		High	363 (51.6%)		184 (61.3%)	
		Severe	121 (46.7%)		209 (65.7%)	
Smoking	Smoker	Low	18 (39.1%)	0.699	11 (37.9%)	0.000
		Mild	53 (34.6%)		31 (38.8%)	
		Average	189 (36.6%)		67 (34.9%)	
		High	261 (37.1%)		151 (50.3%)	
		Severe	78 (41.7%)		182 (57.2%)	
	Non-smoker	Low	28 (60.9%)		18 (62.1%)	
		Mild	100 (65.4%)		49 (61.3%)	
		Average	327 (63.4%)		125 (65.1%)	
		High	443 (62.9%)		149 (49.7%)	
		Severe	109 (58.3%)		136 (42.8%)	

Gender and CSS symptoms were significantly related in both Jordanian and Syrian students. Male Jordanian students had a greater risk of developing CSS symptoms (53.1%), whereas females had a higher likelihood of severe CSS symptoms (54.7%). Syrian students demonstrated a different association, in which females had a higher risk for CSS (54.4%). However, similar to the Jordanian study, it was also observed that Syrian female students tended to score higher toward average to severe, with greater than 50%, compared to males, who tended to score higher toward low to mild, with greater than 50%. Compared to married students, being single increased the likelihood of acquiring CSS symptoms by a high proportion (96.3%) for Syrian students, with 90% or more for mild to severe scores. On the other hand, there was no significant relationship between marital status and CSS among Jordanian students.

The overall CSS score for Jordanian students who lived with five or more people was 50.8%. Students who live with such a large number of people have a higher risk of developing high-to-severe CSS symptoms, with a score of more than 50% for each sub-score, compared to students who live with fewer than five people. Nonetheless, no association was discovered between the number of people living in the household and CSS for Syrian students. In the sample from Jordan, there was a significant association between financial status and CSS score, with students who had a good financial status having a higher chance of developing CSS symptoms, with a total CSS score of 60%. In the sample from Syria, there was no significant relationship between financial level and CSS score.

Both Jordanian and Syrian students showed significant correlations between their field of study and their CSS scores. Students studying at non-medical faculties had a greater probability of acquiring CSS symptoms, with 56.1 % for Jordanian students and 50.8 % for Syrian students. Medical faculty students in both countries tended to score higher toward mild to average sub-scores (50%) and higher. On the other hand, non-medical faculties’ students had a higher tendency for high and severe CSS sub-scores of 50% or more.

Non-smoker Jordanian students had a significantly higher overall CSS score (51.9%) and a significant tendency toward low to average sub-scores of 60% or higher in comparison to smoker students who scored high to severe sub-scores of 50% and above. There were no significant correlations between smoking and CSS scores among Syrian students.

The previous virus infection history has significant correlations with the CSS scores for the students from both countries. Jordanian students who had never been infected with the virus had the highest probability of acquiring CSS symptoms at 46.1%, followed by previously infected students who confirmed their infection by PCR test at 35.8%. Syrian students who had COVID-19 symptoms but were not tested had a greater probability of acquiring CSS symptoms, with an overall CSS score of 52.1%, followed by students who were never infected with the virus, whose overall CSS score was 43.3%. COVID-19 stress syndrome has five domains, each representing a group of symptoms, and each domain has a severity scale ranging from mild to severe, as demonstrated in Table 5.

**Table 5:** COVID-19 stress syndrome domains and distribution between the countries.

COVID-19 stress syndrome domains	Country	Absent	Mild	Moderate	Severe
Danger subscale and contamination subscale	Syria	28%	47%	22%	4%
	Jordan	24%	26%	27%	23%
Socio-economic consequences subscale	Syria	46%	29%	18%	7%
	Jordan	51%	27%	18%	5%
Xenophobia subscale	Syria	66%	22%	10%	2%
	Jordan	44%	23%	21%	13%
Traumatic stress subscale	Syria	87%	10%	3%	0.2%
	Jordan	63%	26%	9%	3%
Compulsive checking subscale	Syria	49%	33%	14%	3%
	Jordan	34%	40%	18%	9%

On the danger and contamination domains, more than half of the Syrian students were experiencing symptoms related to them. In contrast, these symptoms were absent in 24% of Jordanian students and were evenly distributed on the severity scale, with each mild, moderate, and severe representing around 25%. Both Jordanian and Syrian participants significantly experienced symptoms related to the socioeconomic consequences domain, which were seen in around half of the students from both countries. Most of them were of mild presentation, with a percentage of approximately 25% for each country. Similarly, students from both countries have shown mild symptoms related to xenophobia, with a percentage of approximately 25% for each. However, the overall percentage of symptoms related to this domain was 34% among Syrian participants and 56% among Jordanian participants. Eighty-seven percent of the Syrian population has not shown any symptoms related to the posttraumatic domain; similar results were demonstrated in Jordan, where such symptoms were absent in 63% of the total sample. The same pattern of around half of the participants showing symptoms and most of them falling under the mild category was also seen with symptoms related to the compulsive checking domain (Table 5).

## Discussion

The COVID-19 pandemic is going beyond being a physical health catastrophe [17]. Public health strategies dealing with rapidly spreading diseases, such as COVID-19, require strict precautionary measures to control the disease; such measures negatively impact the economy, business, and educational systems, as well as, to some extent, mental health [16,17]. Stress and worry about the consequences of the pandemic are the central features of CSS. A stress scale was developed to measure CSS. Thus, understanding the COVID-19 stress syndrome and assessing its prevalence in an area with a specific culture that might have its influences is paramount to facing the mental and psychological challenges associated with the COVID-19 pandemic.

This study showed a significant correlation between CSS and age among Jordanian students, in which younger students had higher CSS scores than older students. Although this correlation was not established among Syrian students, according to the study by Taylor et al., age and the total score on the CSS scales had a negative correlation [18]. Similar results were also reported by Clemente-Suárez et al., in which younger age groups had a high risk of developing psychiatric symptoms during the COVID-19 pandemic [19]. This relationship can be explained by the fact that Middle Eastern culture considers shifting from school to university a major life event with stressful responsibilities. However, this is not the case in Syria. Although it has the same cultural background as Jordan, it has been witnessing exceptional circumstances that divert the general population’s attention, including students, to the basic life demands and conditions of war. A significant correlation between gender and CSS scores was observed in both countries. Both Jordanian and Syrian female students tended to have severe CSS symptoms. Consistent with our results, the data from the literature suggests that females are more likely to suffer from the significant psychological impact of the outbreak as well as higher levels of stress, anxiety, and depression [7,20]. On the other hand, in a study conducted in Iran, the researchers found no correlation between depression, anxiety, and gender during the COVID-19 pandemic [21]. Another study conducted by Bäuerle et al. indicated that the male gender is a risk factor associated with a higher level of PTSD post-COVID-19 [22]. Our results can be attributed to the differences in the hormonal response to stress along with structural and anatomical differences between males’ and females’ brains [23].

Although there was no significant relationship between marital status and CSS in the Jordan division of the study, single Syrian students had higher CSS scores, indicating that being a single Syrian student is a risk factor for developing the syndrome’s symptoms. Similar to our results from Jordan, marital status was not found to have a significant association with the psychological impacts of the COVID-19 pandemic among Jordanian nurses and the general Chinese population [7,20]. As per our findings from Syria, Monistrol-Mula et al. found that being married decreased the probability of screening positive for anxiety and reduced the risk of anxiety for pre-pandemic mentally disordered patients [24]. Marriage may offer a strong social support system to deal with stressors, which helps explain why marriage has a favorable effect on overall psychological health. The same idea still holds true even though Jordan has not demonstrated the same outcomes, as Jordanian culture may have its own social support system that may include married status but may also extend beyond it, an area of study that requires further research. This study showed a high risk of developing CSS symptoms among Jordanian students living in households of five or more. However, no significant effect on Syrian students was established. Although no studies assessed the relationship between CSS and household size, it was found that COVID-19 stress is highest among individuals who live alone and among those with many other adults [25]. Our findings can be explained by the fact that each additional resident increases the risk of COVID-19 exposure. Moreover, opportunities for alone time and privacy were reduced as many individuals worked and studied from home. Online working and studying were only partially activated in Syria.

Our findings indicated that Jordanian students who were financially stable had a higher risk of experiencing CSS symptoms. But no noticeable impact on Syrian students was found. A Jordanian study demonstrated that students with a medium or low financial situation, compared to economically stable students, tend to be more concerned about educational costs than COVID-19 [26]. However, during the COVID-19 pandemic, it has been reported that individuals with lower economic resources had a more significant burden of depression symptoms [27,28]. Non-medical faculty students in both Syria and Jordan had a greater probability of acquiring CSS symptoms compared with medical faculty students. These results were consistent with a prior study that revealed non-medical students to have a higher prevalence of psychological distress [29]. Additionally, AlJaber found that non-medical students were more likely to experience depression than medical students [30]. On the other hand, several previous studies have illustrated the high level of stress among medical, dental, and nursing students [31,32]. However, during the COVID pandemic, it has been reported that medical students’ anxiety levels decreased while those of non-medical students increased [33]. Several factors can explain our findings: first, the introduction of online learning protects medical students from the perceived risk of COVID-19 as there is no direct contact with patients; second, the variable sources of information about the pandemic among both groups. For instance, limited knowledge about the disease might contribute to negative implications and feelings of fear and anxiety about catching the infection, its symptoms, and its complications [33]. This may be due to several factors, including medical students' being far from the perceived risk of COVID-19 given the introduction of online teaching or the variable sources of information about the pandemic among both groups. Students' fears and anxiety might be reduced by knowledge of the virus. At the same time, an inadequate understanding of COVID-19, its prognosis, transmission, and control measures might contribute to negative implications and a fear of the unknown [33].

The results of our study demonstrated that Jordanian smoker students had a significantly higher CSS score in comparison to non-smokers, and there was no significant correlation between smoking and CSS among Syrian students. It is well established that smoking is one of the coping strategies for stress [34]. Furthermore, consistent with our results, Taylor et al. studied coping strategies with self-isolation and found that people with high CSS scores were more likely to have tried coping strategies [18]. Several studies emphasize this concept, in which they have found that mental distress caused by the effect of precautionary measures formed the primary driving force that led to the significant increase in smoking levels and high rates of ex-smokers relapse, and as a consequence, higher negative mental health impacts were seen with those who smoked more [35,36]. This study is crucial in identifying the root causes of COVID-19 stress syndrome among university students. By reducing the prevalence of this syndrome, we may also mitigate its negative effects, which include academic challenges, social isolation, bullying, and psychological disorders. Using self-reported questionnaires in a cross-sectional study design can limit the validity and reliability of the data, as it can be affected by response bias. Additionally, this type of study design can only suggest correlations or associations but not establish causality. Therefore, further research using different methods is needed to confirm the results and establish causality.

## Conclusions

Beyond just harming people's physical health, the COVID-19 epidemic has had a wide range of detrimental repercussions on the economy, education, and mental health. A stress scale that covers different kinds of concerns and symptoms like DAN, SEC, XEN, TSS, and CHE has been established to gauge the severity of CSS. The development of CSS symptoms has been significantly linked to gender, with higher scores among female pupils. Household size and other characteristics, such as marital status, may also be important. Compared to students in the medical faculty, non-medical faculty students are more likely to have CSS symptoms. Furthermore, Jordanian students' chances of exhibiting CSS symptoms are higher when their financial situation is stable.
